# The gravity-induced re-localization of auxin efflux carrier CsPIN1 in cucumber seedlings: spaceflight experiments for immunohistochemical microscopy

**DOI:** 10.1038/npjmgrav.2016.30

**Published:** 2016-09-15

**Authors:** Chiaki Yamazaki, Nobuharu Fujii, Yutaka Miyazawa, Motoshi Kamada, Haruo Kasahara, Ikuko Osada, Toru Shimazu, Yasuo Fusejima, Akira Higashibata, Takashi Yamazaki, Noriaki Ishioka, Hideyuki Takahashi

**Affiliations:** 1 Graduate School of Life Sciences, Tohoku University, Sendai, Japan; 2 Department of Science and Applications, Japan Space Forum, Tokyo, Japan; 3 Faculty of Science, Yamagata University, Yamagata, Japan; 4 Future Development Division, Advanced Engineering Services Co., Ltd, Tsukuba, Japan; 5 ISS Utilization and Operation Department, Japan Manned Space Systems Co., Tokyo, Japan; 6 JEM Utilization Center, Japan Aerospace Exploration Agency, Tsukuba, Japan; 7 Graduate School of Medicine, Teikyo University, Tokyo, Japan; 8 Institute of Space and Astronautical Science, Japan Aerospace Exploration Agency, Sagamihara, Japan

## Abstract

Reorientation of cucumber seedlings induces re-localization of CsPIN1 auxin efflux carriers in endodermal cells of the transition zone between hypocotyl and roots. This study examined whether the re-localization of CsPIN1 was due to the graviresponse. Immunohistochemical analysis indicated that, when cucumber seedlings were grown entirely under microgravity conditions in space, CsPIN1 in endodermal cells was mainly localized to the cell side parallel to the minor axis of the elliptic cross-section of the transition zone. However, when cucumber seeds were germinated in microgravity for 24 h and then exposed to 1*g* centrifugation in a direction crosswise to the seedling axis for 2 h in space, CsPIN1 was re-localized to the bottom of endodermal cells of the transition zone. These results reveal that the localization of CsPIN1 in endodermal cells changes in response to gravity. Furthermore, our results suggest that the endodermal cell layer becomes a canal by which auxin is laterally transported from the upper to the lower flank in response to gravity. The graviresponse-regulated re-localization of CsPIN1 could be responsible for the decrease in auxin level, and thus for the suppression of peg formation, on the upper side of the transition zone in horizontally placed seedlings of cucumber.

## Introduction

Plants respond to gravity by changing their growth orientation and morphology.^1,2^ The formation of a specialized protuberance, the peg, in cucurbitaceous seedlings is a unique gravimorphogenesis.^2,3^ When cucumber seeds (*Cucumis sativus* L.) are placed in a horizontal position and allowed to germinate, a peg forms on the lower side of the transition zone between the hypocotyl and the root. The peg anchors the lower seed coat in soil so that the elongation of the hypocotyl pulls the cotyledons out of the seed coat. The peg therefore facilitates the emergence of seedlings from the hard seed coat. Cucumber seedlings have the potential to develop a peg on each side of the transition zone as, when seeds are placed before germination in a vertical position with the radicles pointing down or under microgravity conditions, a peg develops on each side.^4^ However, peg formation on the upper side of the transition zone is suppressed in response to gravity when the seedlings are grown in a horizontal position on the ground.^4^

A phytohormone, auxin, has an important role in the lateral placement of peg formation in the transition zone.^2,3^ Application of indole-3-acetic acid (IAA), the main auxin in plants, promotes peg development, and its endogenous concentration is significantly reduced in the peg-suppressed side (the upper side) of the transition zone.^2,5,6^ Furthermore, treatment of seedlings with the auxin transport inhibitors 2,3,5-triiodobenzoic acid or 9-hydroxyfluorene-9-carboxylic acid blocks the suppression of peg formation on the upper side and causes the development of a peg on each side of the transition zone, even when seedlings are germinated in a horizontal position.^7^ This suggests that gravity-modified transport of auxin is required for the differential decrease in auxin level on the upper side of the transition zone in cucumber seedlings grown in a horizontal position. By contrast, the lower side of the transition zone can maintain the higher auxin level required for peg formation.^5,7^

Plasma membrane-localized auxin efflux proteins of the PIN-FORMED (PIN) and P-glycoprotein families facilitate the transport of auxin.^8–10^ In particular, the polarity of PIN localization corresponds to the direction of auxin transport.^11,12^ In Arabidopsis (*Arabidopsis thaliana*), AtPIN3 and AtPIN7 proteins that are expressed in gravisensing columella cells, respond to reorientation of roots by changing their localization to the side that has newly become the lower side of the cells.^13,14^ Similarly, reorientation of Arabidopsis hypocotyls induces the re-localization of AtPIN3 to the lower side of gravisensing endodermal cells.^15^ In cucumber, we have shown that reorientation of seedlings from a vertical position to a horizontal position induced changes in CsPIN1 localization in endodermis as well as asymmetric redistribution of auxin within 30 min of reorientation in the transition zone.^6^ These observations led to the hypothesis that the change in CsPIN1 localization in the endodermis following the reorientation influences auxin transport through the endodermis, which results in asymmetric auxin distribution in the transition zone.^6^ However, because these studies of CsPIN1 and AtPIN3 localization were studied using longitudinal sections,^6,13,15^ the pathway of auxin transport via endodermal layers has been poorly understood not only in cucumber but also in other plant species including Arabidopsis. Furthermore, the gravity-inducible change in PIN proteins remains to be verified in microgravity.

Here we examined the CsPIN1 localization using the cross-sections of the transition zone of cucumber seedlings grown under microgravity conditions. The results showed that endodermal cells re-localize CsPIN1 due to gravistimualtion and laterally transport auxin from the upper to the lower flank, which explains the redistribution of auxin responsible for the lateral placement of peg formation in cucumber seedlings.

## Results

### Growth and morphogenesis of cucumber seedlings in microgravity

We previously reported that, when 24-h-old cucumber seedlings grown vertically were placed in a horizontal position, the localization of CsPIN1 in the endodermal cell layer of the transition zone changed.^6^ We conducted spaceflight experiments on the International Space Station (ISS) to investigate the effects of gravistimulation on CsPIN1 localization.

Before spaceflight experiments, we examined the effects of gravistimulation on peg formation using 24-h-old cucumber seedlings in ground control experiments. For this experiments, we used a two-axis clinostat to rotate cucumber seedlings through three dimensions, because clinorotation randomizing the position of plants against gravity direction is used as an analog for some plant responses under microgravity conditions.^16–18^ When cucumber seedlings were grown under clinorotated conditions for 24 h and then either maintained continuously on the rotating clinostat or transferred to stationary conditions in a vertical position for 48 h, over half of the seedlings developed a peg on each side of the transition zone (Table 1). On the other hand, when cucumber seedlings were clinorotated for 24 h after seed imbibition and then gravistimulated by placing them in a horizontal position, most seedlings developed a peg only on the lower side of the transition zone after 48 h of growth (Table 1). It was shown that a peg formed on the new lower side but did not form on the new upper side when the horizontally grown ~24-h-old cucumber seedlings were inverted up-side down.^3^ These results suggest that 24-h-old seedlings are still able to respond to gravity in determining the position of peg formation. To examine the gravity-inducible re-localization of CsPIN1, therefore, we grew cucumber seedlings in space microgravity for 24 h and then exposed them to three different gravitational conditions for 2 h as follows: (i) a continued microgravity environment, (ii) a 1*g* centrifugal force in a longitudinal direction to the axis of hypocotyl–root of the seedling, and (iii) a 1*g* centrifugal force in a crosswise direction to the axis of hypocotyl–root of the seedling.

**Figure 1:**
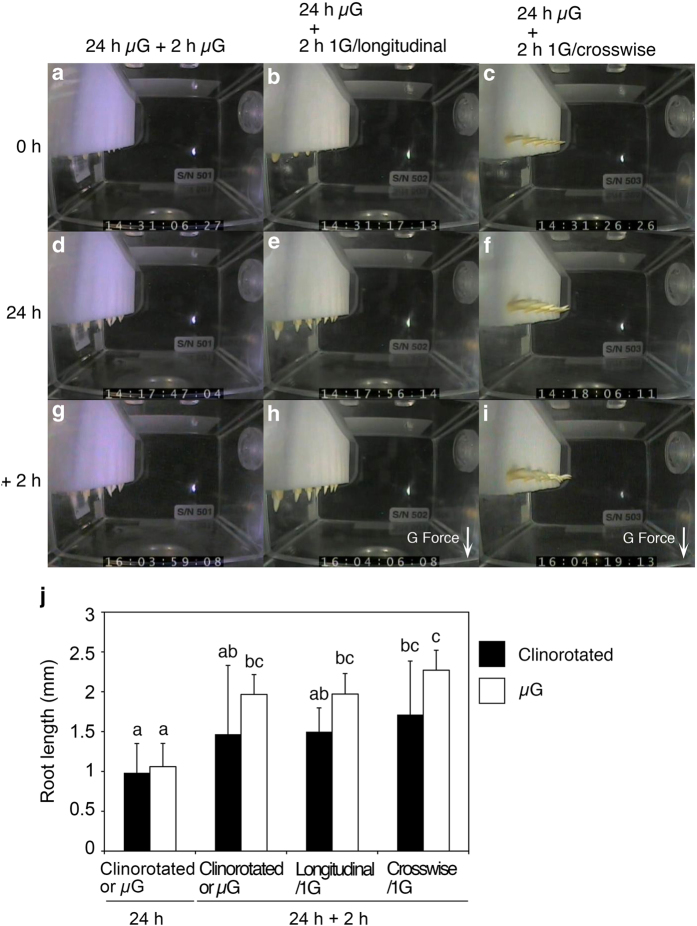
Seed germination and seedling growth of cucumber in space. The water-absorbent plastic foam in the container was supplied with water (**a**–**c**), and then germinating seedlings were grown in the microgravity compartment of CBEF for 24 h (**d**–**f**). Then, cucumber seedlings were either maintained in microgravity (**g**), or exposed to a 1*g* centrifugal force applied longitudinally (**h**) or in a crosswise direction (**i**) for a further 2 h. Photographs of the seedlings were taken on the ISS before fixation. After storage in fixative for ~1 month in space and returning the spaceflight samples to Earth, the root lengths of these seedlings were measured (**j**) and these seedlings were analyzed immunohistochemically. Each datum represents the mean±s.d. of seven cucumber seedlings, and different letters indicate statistically significant differences between groups at *P*<0.05 using Tukey’s method (**j**). Arrow (G), the direction of centrifugal force. CBEF, Cell Biology Experiment Facility; ISS, International Space Station.

The germination rate of cucumber seeds in our spaceflight experiments was 100% (Figure 1a–i). The roots of seedlings grown in microgravity grew in various directions slightly deviating from the seedling axis (Figure 1d,g). On the other hand, roots grew straight and followed the direction of gravitational force when 1*g* was applied longitudinally for 2 h (Figure 1h). Similarly, when seedlings were exposed to 1*g* in the crosswise direction to the axis of hypocotyl–root for 2 h their roots bent downward, due to the 1*g* vector generated by centrifugation (Figure 1i). We compared the root lengths of seedlings grown in microgravity with those of clinorotated seedlings on the ground. Although root length tended to be slightly longer in space, there was statistically no differences observed and there appeared to be continuously growing for 2 h treatments after 24 h of germination (Figure 1j).

### Effect of centrifugal 1*g* on CsPIN1 localization in cucumber seedlings grown in microgravity

**Figure 2:**
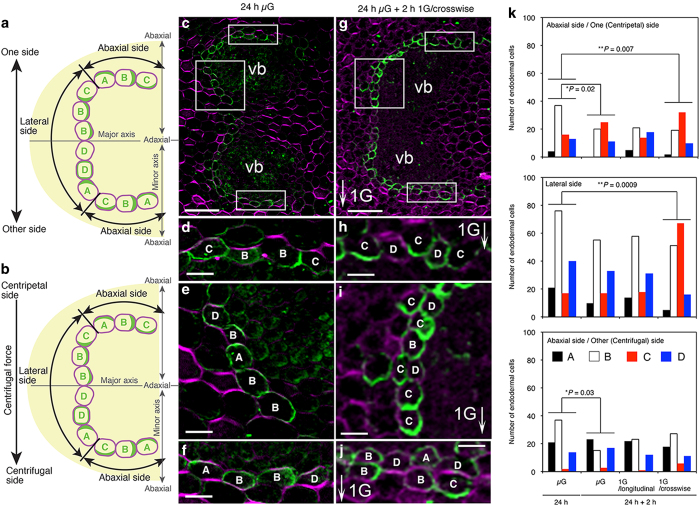
CsPIN1 localization in the cross-sections obtained from the transition zone of cucumber seedlings grown in space. Schematic representations for the classification of the endodermal cells based on CsPIN1 localization patterns in the transition zone are shown (**a**, **b**). The types of endodermal cells of the cucumber seedlings grown in microgravity or exposed to 1*g* centrifugal force in the longitudinal direction were classified according to **a**, whereas those of the cucumber seedlings exposed to 1*g* centrifugal force in the crosswise direction were classified according to **b**. Four types of endodermal cells with different localization of CsPIN1 (green crescent shaped) are indicated by the capital letters, A–D, in each cell. Micrographs show CsPIN1 localization (**c**–**j**); a half of the cross-section of the transition zone in seedling grown in microgravity for 24 h (**c**) and in seedling exposed to 1*g* for 2 h in a direction crosswise to seedling axis following 24-h microgravity (**g**). The top, left, and bottom boxes drawn by white lines in **c** were enlarged in **d**–**f**, respectively. The top, left, and bottom boxes drawn by white lines in **g** were enlarged in **h**–**j**, respectively. Signals for antibody staining appear green. Staining of the cell wall by Fluorescent Brightener 28 appears magenta. vb, vascular bundle; scale bar=100 μm (**c**, **g**); 25 μm (**d**–**f** and **h**–**j**); arrow (G), direction of gravitational force. Cell types, A–D, are distinguished in **d**–**f** and **h**–**j**. The numbers of endodermal cells classified into each type of CsPIN1 localization in each endodermal region observed are shown (**k**). Seedlings were grown in microgravity for 24 h (24 h μG), before being either continuously grown in microgravity (μG), or exposed to 1*g* in a direction longitudinal (1G/longitudinal) or crosswise (1G/crosswise) to seedling axis for a further 2 h. Black, white, red, and blue bars indicate cell types A, B, C, and D, respectively. Each datum represents the mean±s.d. of 6–10 images obtained from three to five cucumber seedlings. *Single asterisks and **double asterisks indicate statistically significant differences when the number of endodermal cells that were categorized in the control (24 h μG) were compared with those of the others using Fisher’s exact test at *P<*0.05 and *P*<0.01, respectively.

We immunohistochemically stained cross-sections of the transition zones of cucumber seedlings with anti-CsPIN1 antibodies. To characterize CsPIN1 localization in the endodermal cells of the transition zone, we divided the endodermal cell layer, which was observed in half of the cross-section of the transition zone, into the three regions shown in Figure 2a,b. The cross-section of the transition zone appeared elliptical in shape. The half of the transition zone of a non-gravistimulated seedling was further divided into halves (labeled ‘one side’ and ‘other side’) by the major axis of the elliptical cross-section (Figure 2a). Both halves included endodermal cells situated in the ‘abaxial’ (in the plane of the cotyledon side) and ‘lateral’ (in the plane of the non-cotyledon side) regions of the transition zone. Likewise, in the transition zone of seedlings exposed to 1*g* centrifugal force applied in the crosswise direction to the axis of hypocotyl–root, the endodermal cell layers were situated in both the ‘centripetal’ and ‘centrifugal’ regions divided by the major axis of the cross-section (Figure 2b). We further categorized the types of endodermal cells with reference to the polarized localization of CsPIN1 signals within the cells. In the transition zones of non-gravistimulated seedlings grown either in microgravity or exposed to 1*g* centrifugal force applied in the longitudinal direction to the axis of hypocotyl–root (Figure 2a), CsPIN1 in type A endodermal cells localized to the direction of the abaxial/one side; CsPIN1 in type B cells localized to the cell side parallel to the minor axis of the elliptic cross-section; and CsPIN1 in type C cells localized to the direction of the abaxial/other side. Endodermal cells in which the CsPIN1 localization pattern was not distinct were classified as type D. The type D included cells that show CsPIN1 on all around the plasma membrane or no CsPIN1 signals at all. In the transition zones of seedlings exposed to 1*g* centrifugal force applied in the crosswise direction to the axis of hypocotyl–root (Figure 2b), CsPIN1 in type A and C endodermal cells localized to the direction of the ‘centripetal’ and ‘centrifugal’ sides, respectively, and in type B cells CsPIN1 localized to the cell side parallel to the minor axis of the elliptic cross-section; again, cells other than types A, B, and C were classified as type D.

When cucumber seedlings were grown in microgravity for 24 h, CsPIN1 signals were detected at the cell side parallel to the minor axis of the cross-section of the transition zone (Figure 2c–f,k). When 24-h-old cucumber seedlings grown in microgravity were incubated for a further 2 h in microgravity, the pattern of CsPIN1 in the abaxial sides but not in the lateral side slightly differed from that of 24-h-old cucumber seedlings grown in microgravity at *P*<0.05 level (Figure 2k). In cucumber seedlings grown for a further 2 h with 1*g* centrifugal force applied in a longitudinal direction to the axis of hypocotyl–root, the pattern of CsPIN1 in the abaxial side did not differ from that of 24-h-old cucumber seedlings grown in microgravity or that of the seedlings grown for further 2 h in a vertical position (Figure 2k). However, when 24-h-old cucumber seedlings were exposed to 1*g* centrifugal force applied in a crosswise direction to the axis of hypocotyl–root for 2 h, the pattern of CsPIN1 localization in the abaxial/centripetal side and the lateral side significantly differed from that of 24-h-old cucumber seedlings grown in microgravity at *P*<0.01 level (Figure 2g–j,k). This re-localization of CsPIN1 due to gravistimulation was much pronounced in the lateral side (Figure 2g,i,k). The localization pattern of CsPIN1 in the transition zone in cucumber seedlings after exposing to 1*g* centrifugal force for 2 h was characterized as an increased number of type C cells that localized CsPIN1 in endodermal cells at the abaxial/centripetal and lateral sides (Figure 2g–i,k). In the abaxial/centrifugal side of the transition zone of cucumber seedlings exposed to 1*g* centrifugal force for 2 h, the pattern of CsPIN1 localization did not differ from that of 24-h-old cucumber seedlings grown in microgravity (Figure 2g,j,k). These results suggested that gravistimulation to the space-grown cucumber seedlings with a 1*g* centrifugal force applied in the crosswise direction induced a change in the localization of CsPIN1 and caused it to accumulate at the bottom of the gravisensing endodermal cells in abaxial/centripetal and lateral sides of the transition zone.

## Discussion

Previously, we reported that CsPIN1 in the endodermal cells re-localize to the bottom side upon reorientation of the seedlings, which is observable on the upper endodermal cells in the transition zone between the hypocotyl and the root of the horizontally placed seedlings of cucumber.^6^ This result suggested that the pronounced auxin efflux due to CsPIN1 causes a decrease in auxin level in the upper side of the transition zone.^6^ Here our spaceflight study demonstrate that CsPIN1 re-localization is a graviresponse, which could results in auxin redistribution in the transition zone of cucumber seedlings. In addition, the results of this study reveal that endodermal cell layer with the polarized CsPIN1 localization could become a canal for the lateral auxin transport from the upper to the lower flank of the gravistimulated transition zone.

Auxin distribution in Arabidopsis is mainly regulated by the directional transport of auxin as follows: auxin synthesized in the shoot apex is transported toward the root tip through vascular bundle cells by basipetally localized auxin efflux carriers, such as AtPIN1.^11^ Root columella cells expressing the auxin efflux carriers AtPIN3 and AtPIN7 transport auxin toward the lateral root cap cells.^13,14^ Epidermal cells, which accumulate the AtPIN2 auxin efflux carrier, transport auxin from the lateral root caps to the elongation zone, in which bending occurs due to a differential growth.^12^ The reorientation of roots from a vertical to a horizontal position induces the re-localization of AtPIN3 and AtPIN7 to the lower side of the columella cells.^13,14,19^ As a result, auxin transport occurs asymmetrically from the columella cells to the lateral root cap cells and then to the lower flank of the elongation zone in a horizontal position. This mechanism provides a good explanation for root gravitropism known as the Cholodny and Went hypothesis, which holds that gravitropic curvature of a growing plant organ depends on asymmetric auxin distribution.^20,21^

In contrast to roots, which sense gravity via columella cells, lateral auxin transport in hypocotyls and shoots, in which endodermal cells undergo gravistimulation, is poorly understood. Reorientation of cucumber seedlings from a vertical to a horizontal position increases CsPIN1 accumulation on the lower side of endodermal cells in the upper endodermis of the transition zone,^6^ and in horizontally oriented hypocotyls of Arabidopsis, AtPIN3 accumulation decreases in the outer side of the upper endodermal cells and in the inner side of the lower endodermal cells.^15^ This suggests that endodermal cells in the upper endodermis prevent auxin transport from vascular cells to cortical cells in the transition zone of cucumber and in Arabidopsis hypocotyls. They could also promote auxin transport from the endodermis to vascular tissue in the upper flank of the gravistimulated tissues. In the lower side of the gravistimulated hypocotyls of Arabidopsis, however, localization of AtPIN3 at the lower endodermal cells can facilitate transport of auxin from endodermal cells to cortical cells.^15^ These models were based on the observation of the longitudinal sections and two-dimensional images of the transition zone and hypocotyls.^6,15^ However, the re-localization of auxin efflux carriers including AtPIN3 in endodermal layers in response to gravistimulation on the cross-sections has not been shown before. Our results using cross-sections showed that, following horizontal placement of cucumber seedlings, the number of endodermal cells in which CsPIN1 was localized at the lower side of the cells in the upper and lateral endodermis (Figure 2; abaxial/centripetal and lateral sides) of the transition zone increased. This observation implies that endodermal cells laterally transport auxin to enable a greater accumulation of auxin in the lower flank of the transition zone.

**Figure 3:**
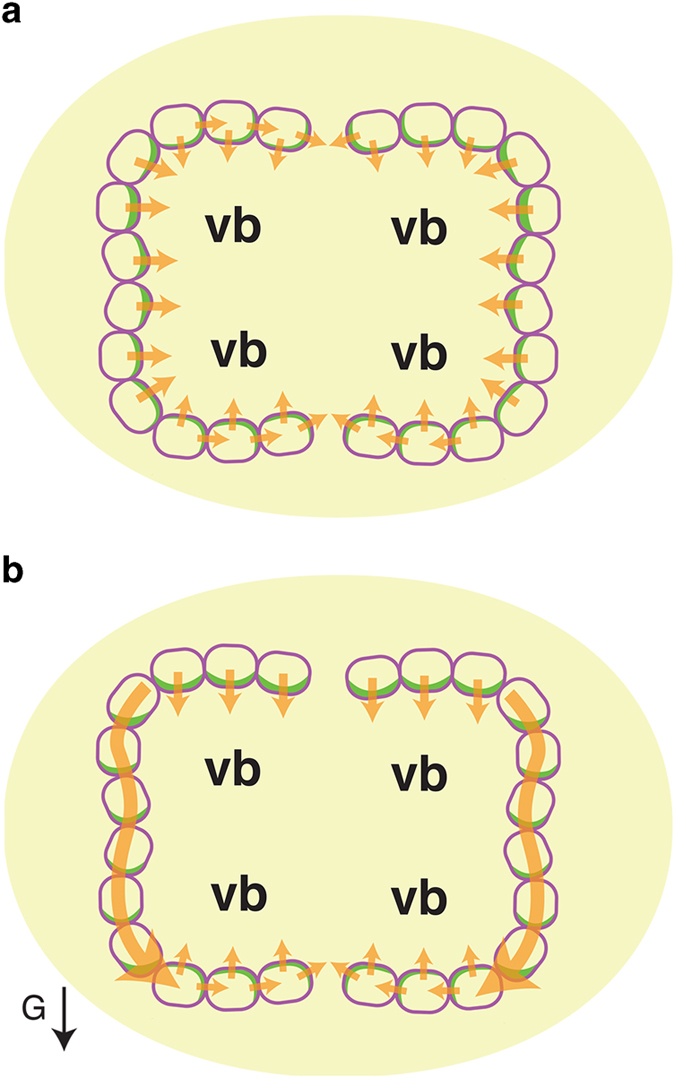
A model for the graviresponse induction of asymmetric auxin distribution by CsPIN1-mediated auxin transport in the transition zone of the cucumber seedlings. Endodermal cells and the localized CsPIN1 auxin efflux carriers are shown by the purple line and green color, respectively. vb, vascular bundle; orange arrow, direction of auxin flux; arrow (G), the direction of gravitational force. Auxin synthesized in cotyledons or the shoot apical meristem is transported toward the root.^3^ CsPIN1 is an auxin efflux protein basipetally localized in cells of the vascular bundles and contributes to this directional auxin transport.^6^ When cucumber seedlings are grown in a vertical position on the ground or in microgravity, auxin can be prevented from moving from vascular tissue to the cortex by localization of CsPIN1 along the minor axis of endodermal cells in the transition zone (**a**). When cucumber seedlings are reoriented to the horizontal position, CsPIN1 re-localizes to the lower side of the endodermal cells in the lateral endodermis of the transition zone, and thus the endodermal cell layer laterally transports auxin from the upper to the lower side (**b**). Localization of CsPIN1 may therefore have a role in facilitating the decrease in auxin levels on the upper side of the transition zone in gravistimulated cucumber seedlings, and thus in suppressing peg formation in this region.

When cucumber seeds are placed and grown either in a vertical position or in microgravity conditions, a peg develops on each side of the transition zone.^4^ We observed that, in the transition zone of cucumber seedlings grown in a vertical position or in microgravity, CsPIN1 signals in the abaxial endodermal cells were detected at the adaxial side and the cell side parallel to the minor axis of the cross-section of the transition zone (Figure 3a). Under these growth conditions, a peg develops on each side of the transition zone, and thus this pattern of CsPIN1 localization does not affect the symmetrical auxin distribution and cannot cause the reduction of auxin required to suppress peg formation. This conclusion was consistent with that auxin-inducible *CsIAA1* messenger RNA symmetrically accumulated in the transition zone of cucumber seedlings grown in microgravity.^5^ These results of expression of auxin-inducible *CsIAA1* gene, the localization of CsPIN1 proteins and peg formation suggest that the cucumber seedlings grown in a vertical position and those grown in microgravity conditions possess physiologically a similar status. Recently, it has been described that the expression of auxin reporter gene (*pDR5r::GFP*) in Arabidopsis grown in microgravity was identical to that of the ground control, although the expression of cytokinin reporter gene (*pARR5::GFP*) in microgravity differed from that on the ground.^22^ Therefore, the responses of plants to 1*g* would not affect auxin status but those to the direction of gravity would affect the PINs’ localization and then auxin distribution.

The present study suggests the endodermal cell layers of the transition zone become a canal for auxin transport from the upper to the lower side of the transition zone (Figure 3b). This lateral auxin transport pathway composed of endodermal layers may induce an asymmetric distribution of auxin across the transition zone of cucumber seedlings. In addition, localization of CsPIN1 to the lower side of endodermal cells on the upper side of the transition zone could contribute to the graviresponse negatively regulating peg formation by preventing auxin transport from vascular tissue to the cortex and epidermis and/or by removing auxin from the cortex and epidermis. This model is probable, because asymmetric expression of an auxin-inducible gene, *CsIAA1*, is detected in the epidermis and cortex across the transition zone.^5,23,24^ Regulation of auxin levels in the cortex and epidermis in this way may be an important factor responsible for the lateral placement of peg formation in cucumber seedlings.

In conclusion, our spaceflight experiments demonstrate that gravistimulation induces re-localization of CsPIN1 auxin efflux carriers in endodermal cells in the transition zone of cucumber seedlings. Our results further suggest that re-localization of CsPIN1 auxin efflux carriers in endodermis due to gravistimulation enables endodermal cell layers to transport auxin from the upper to the lower side of the transition zone of cucumber seedlings.

## Materials and methods

### Plant materials and fixation

On the ground, seven seeds of *Cucumis sativus* L. cv. Shinfushinarijibai (Watanabe Seed Co., Kogota, Miyagi, Japan) were vertically or horizontally inserted into a crack within a water-absorbent plastic foam placed in a plastic container, as shown in Supplementary Figure S1. A metallic pipe containing small holes and connected to a port for the injection of water was inserted into the water-absorbent plastic foam (49×15×10 mm). The plastic containers holding the seeds, together with other instrumentation, were loaded into the STS-133 space shuttle, Discovery, and launched from the Kennedy Space Center on 24 February 2011. While in orbit on the ISS, a plastic syringe fitted with a tap and a tube for drawing water from the water reservoir was connected to the port of the plastic container. Pushing a plunger supplied 10 ml of distilled water to the foam so that imbibition of cucumber seeds was initiated. The plastic container was placed in Measurement Experiment Unit B, which is fitted with a charge-coupled device camera, light-emitting diode lamps, and a temperature recorder. The Measurement Experiment Unit B was then placed in the Cell Biology Experiment Facility, an incubator unit consisting of a microgravity compartment and a centrifuge compartment, which can provide centrifugal force from 0.1 to 2.0 *g*. Cucumber seeds were allowed to germinate and the resulting seedlings were grown in microgravity or exposed to 1*g* centrifugation at 25±1 °C in the dark. After incubation, the seedlings were photographed and fixed with a fixative, acetic acid:ethanol:distilled water (5:63:32), using the Kennedy Space Center Fixation Tube (KFT). For fixation in microgravity, each piece of water-absorbent plastic foam holding seven cucumber seedlings was detached from its container, put into the KFT containing the fixative, and stored at 4 °C in the Minus Eighty Degree Celsius Laboratory Freezer for ISS until they were returned to Earth by the space shuttle *Atlantis* (STS-135) and sent to our laboratory. The seedlings were kept refrigerated at 4 °C during shipping and were stored in fixative for ~1 month. After the KFT was opened, the samples were infiltrated with newly prepared fixative, acetic acid: ethanol: distilled water (5:63:32), and stored overnight at 4 °C.

For ground experiments, the plastic containers were placed on the two-axis clinostat and three dimensionally rotated at 2 r.p.m. for 24 h.^16–18^ For gravistimulation of the seedlings, the containers were detached from the clinostat and placed on the ground ensuring that seedlings were placed either in a vertical or a horizontal position for 2 h or 48 h. For non-gravistimulation, seedlings were further clinorotated for 2 h or 48 h. These experiments were performed at 25±1 °C in the dark. After incubation, the samples were photographed.

The length of roots of cucumber seedlings was measured using the photograph images and ImageJ ver. 1.42 software (NIH, Bethesda, MD, USA).

### Immunohistochemical analysis

Histochemical staining for immunolocalization of CsPIN1 was performed as previously described,^6^ with some modifications. Although previously ethanol:chloroform:acetic acid (6:3:1) was used as a fixative for immunohistochemical analysis of CsPIN1, this was changed to acetic acid:ethanol:distilled water (5:63:32) because of the safety regulations on the ISS. The hypocotyl side of the transition zone contains four vascular strands and develops endodermal layers around each vascular strand whereas endodermal layers that surround two vascular strands fuse in the root-side transition zone. CsPIN1 signals in seedlings fixed by the two fixatives were compared in advance: the signal intensity on the hypocotyl side of the transition zone was much stronger in seedlings fixed with ethanol:chloroform:acetic acid (6:3:1) than in those fixed with acetic acid:ethanol:distilled water (5:63:32). CsPIN1 signal intensities on the root side of the transition zone, however, were similar in seedlings fixed with the two fixatives. Thus, we analyzed the root side of the transition zone in this study. Fixed segments were used for immunohistochemical analysis as described.^6^ To evaluate localization of CsPIN1 in endodermal cells of the transition zone, the types of endodermal cells were classified based on CsPIN1 localization in 6–10 images in half of the cross-section that were obtained from three to five cucumber seedlings (both halves of the cross-sections were used). The numbers of each cell type were counted by single-blind assay as follows. The file names of images of immunohistochemistry were changed, and the changes were recorded. Then, one who did not know the change classified the endodermal cells by CsPIN1 localization. After classification, the numbers of classified cells in each experiment were counted based on the records.

### Statistical analysis

Tukey’s method, using KaleidaGraph Ver. 4.1J (Synergy Software, Reading, PA, USA), was adopted to analyze the root lengths of cucumber seedlings according to manufacturer’s instrument. For analyses of the effects of gravistimulation and clinorotation on peg formation and of the effects of gravistimulation on CsPIN1 localization, Fisher’s exact (two-sided) test was performed using ‘fisher.test’ that was a default command in R, version 3.1.3 (http://www.r-project.org; R Development Core Team, Boston, MA).

## Figures and Tables

**Table 1:** Effect of gravistimulation on the frequency of peg formation in clinorotated cucumber seedlings^a^

*Growth conditions*	*Number of seedlings*		*Total*
	*With one peg*	*With two pegs*	
Clinorotation for 72 h	13	15	28
Clinorotation for 24 h+vertical for 48 h	10	17	27
Clinorotation for 24 h+horizontal for 48 h	27*	1*	28

a Growing cucumber seedlings were rotated on the clinostat for 24 h and then placed in a vertical or horizontal position for 48 h. As a control, growing cucumber seedlings were rotated on the clinostat for 72 h. Values indicate the number of seedlings that formed one or two peg(s). Experiments (*n*=6–7) were repeated four times. Asterisk indicates a statistically significant difference when compared with clinorotation controls (Fisher’s exact test, *P*<0.01).
