# Supplementary material for: The gravity-induced re-localization of auxin efflux carrier CsPIN1 in cucumber seedlings: spaceflight experiments for immunohistochemical microscopy
Source: NPJ Microgravity. 2016 Sep 15;2:16030–. doi: 10.1038/npjmgrav.2016.30 (PMC5515524; doi:10.1038/npjmgrav.2016.30)
Supplement: Supplementary Figure S1 Legends [file npjmgrav201630-s1.doc]

Supplementary Figure S1. Seeds were inserted into the plastic foam as shown in a,c to grow seedlings in microgravity or to expose seedlings to 1G centrifugal force applied longitudinally in space, and as shown in b to expose seedlings to 1G centrifugal force in the crosswise direction.
