# Supplementary figures and images for: The gravity-induced re-localization of auxin efflux carrier CsPIN1 in cucumber seedlings: spaceflight experiments for immunohistochemical microscopy
Source: NPJ Microgravity. 2016 Sep 15;2:16030–. doi: 10.1038/npjmgrav.2016.30 (PMC5515524; doi:10.1038/npjmgrav.2016.30)

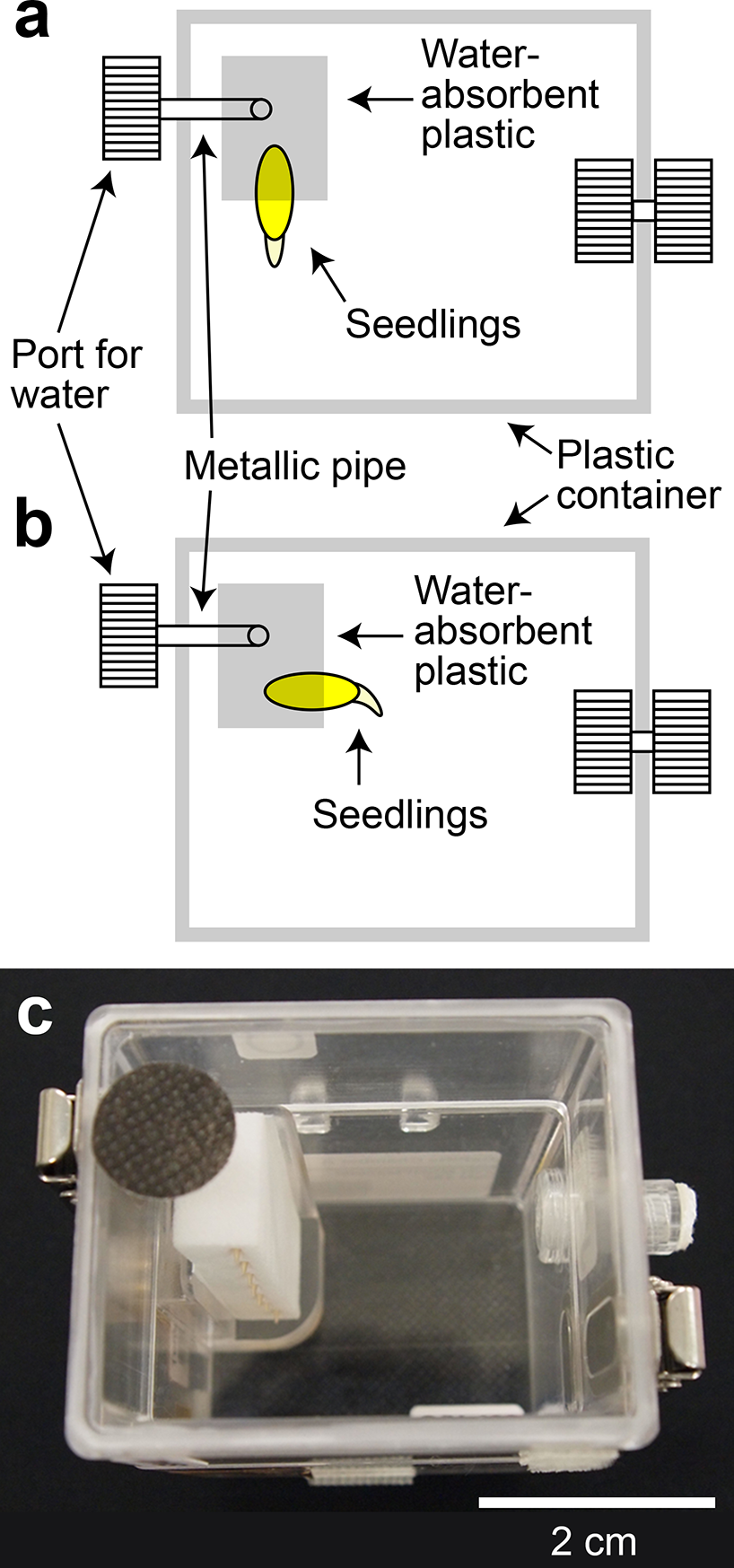

Supplement: Supplementary Figure S1 [file npjmgrav201630-s2.tiff]
